# Mapping effective connectivity within cortical networks with diffuse optical tomography

**DOI:** 10.1117/1.NPh.4.4.041402

**Published:** 2017-07-21

**Authors:** Mahlega S. Hassanpour, Adam T. Eggebrecht, Jonathan E. Peelle, Joseph P. Culver

**Affiliations:** a Washington University in St. Louis, Department of Physics, St. Louis, Missouri, United States; b Washington University in St. Louis, Department of Radiology, St. Louis, Missouri, United States; c Washington University in St. Louis, Department of Otolaryngology, St. Louis, Missouri, United States; d Washington University in St. Louis, Department of Biomedical Engineering, St. Louis, Missouri, United States

**Keywords:** diffuse optical tomography, effective connectivity, psychophysiological interaction, speech comprehension

## Abstract

Understanding how cortical networks interact in response to task demands is important both for providing insight into the brain’s processing architecture and for managing neurological diseases and mental disorders. High-density diffuse optical tomography (HD-DOT) is a neuroimaging technique that offers the significant advantages of having a naturalistic, acoustically controllable environment and being compatible with metal implants, neither of which is possible with functional magnetic resonance imaging. We used HD-DOT to study the effective connectivity and assess the modulatory effects of speech intelligibility and syntactic complexity on functional connections within the cortical speech network. To accomplish this, we extend the use of a generalized psychophysiological interaction (PPI) analysis framework. In particular, we apply PPI methods to event-related HD-DOT recordings of cortical oxyhemoglobin activity during auditory sentence processing. We evaluate multiple approaches for selecting cortical regions of interest and for modeling interactions among these regions. Our results show that using subject-based regions has minimal effect on group-level connectivity maps. We also demonstrate that incorporating an interaction model based on estimated neural activity results in significantly stronger effective connectivity. Taken together our findings support the use of HD-DOT with PPI methods for noninvasively studying task-related modulations of functional connectivity.

## 1 Introduction

Functional neuroimaging has provided substantial insight into how distributed dynamic neural processes are related in space and time. Collections of brain regions implicated in task-based or resting-state studies constitute functionally integrated systems of neural networks with properties that have been shown to be remarkably consistent across healthy participants.^1^^–^^3^ Importantly, disruptions or alterations of these functional networks have been shown to correlate with the stage of multiple neurodevelopmental and neurodegenerative conditions such as Autism and Alzheimer’s diseases.^4^^–^^8^ Mapping functional brain networks can potentially aid in early prediction of these conditions and could facilitate the optimization of interventions and tracking of treatment efficacy. However, technical limitations such as a fixed scanner, contraindication with metal implants, loud scanner noise, and emotional distress associated with the fear of small spaces limit the clinical and translational applications of functional magnetic resonance imaging (fMRI) for many types of diagnostic and prognostic studies.

An emerging optical neuroimaging technique, high-density diffuse optical tomography (HD-DOT), has the potential to be used as a surrogate to fMRI in many circumstances. HD-DOT provides a more naturalistic imaging setting where the acoustic environment is highly controllable. It is metal-compatible, cap-based imaging that samples the brain’s hemodynamic response with a relatively high temporal resolution [compared to time course of the hemodynamic response function (HRF)] and provides fMRI-comparable anatomical localization at the gyral level.^9^ To date, DOT studies have been mainly focused on investigating functional segregation (i.e., activity in specific brain regions related to a particular task)^10^^–^^12^ or evaluating resting state functional connectivity (i.e., correlations among intrinsic activities of remote brain regions).^13^^,^^14^ Excluding topographic studies using functional near-infrared spectroscopy,^15^^,^^16^ no previous studies have used optical neuroimaging to map effective connectivity within the brain (i.e., the influence that one brain region exerts over another during a task performance^17^) (Note that functional connectivity is a statistical relationship between two time series, whereas effective connectivity refers to the influence one system exerts over another, i.e., it implies a directionality to the relationship; see Ref. 18).

In the fMRI literature, psychophysiological interaction (PPI) analysis has been among the most promising techniques for examining effective connectivity.^19^^–^^21^ PPI analyses provide estimates of effective connectivity patterns by modeling activity in brain regions in terms of the interactions among the input from a seed brain region of interest (ROI) (physiological component) and the demand imposed by a cognitive process (psychological component). In this study, we use a generalized PPI (gPPI) technique^19^ to investigate functional integration during speech comprehension. We examine different strategies for selecting seeds and modeling interregional connections of HD-DOT data.

One challenge facing all effective connectivity studies is the selection of the seed ROIs. Methods for selecting seeds vary across studies, and currently there is no established standard approach for seed selection. Seeds can be defined in at least four different ways:

1. Using anatomical landmarks or coordinates, as when there is a strong hypothesis about a particular anatomical region,^11^^–^^13^
2. Using regions with the strongest task-dependent response in group results,^22^^–^^24^
3. Using peaks from each individual’s task-dependent response, which may help account for interindividual differences in functional anatomy,^25^ or
4. Using a combination of these methods.^26^^–^^28^

In the current study, we investigate the degree to which different seed selection methods impact estimated effective connectivity.

Because interactions in the brain occur at the neural level (rather than the hemodynamic level), an accurate model of neural interactions requires the neural signal, whereas HD-DOT provides only an indirect measure of the neural activity (as does the blood-oxygenation level-dependent signal measured with fMRI). Prior applications of PPI in fMRI data have suggested calculating interactions on the estimated neural activity based on a deconvolution approach.^21^ Here, we examined whether an interaction model based on deconvolution of a canonical HRF from the hemodynamic activity at the seed location can increase the statistical significance of measures of effective connectivity relative to a no deconvolution routine (where interactions are simply modeled at hemodynamic level).

To examine these methodological decisions and explore the use of PPI analyses in HD-DOT, we used data from an auditory sentence comprehension task. We selected speech comprehension because of its distributed and hierarchical organization in the brain that requires interaction among discrete cortical regions, it is a behavior that is frequently of interest in clinical populations,^29^^–^^31^ and it is well suited for optical neuroimaging.^32^ Using sentences with different levels of syntactic complexity, we examine how demands made by syntactic processes are manifested in the patterns of interregional connections. We hypothesized that processing sentences with increasing levels of linguistic challenge would result in increased effective connectivity within the speech network.

## 2 Materials and Methods

Herein are new analyses of data that were presented in a previous paper.^12^

### 2.1 Participants

Participants were 10 healthy right-handed native English speakers (ages 20 to 30 years, mean=27.6, STD=3.3, 6 female). All had normal hearing by self-report and no history of neurological or psychiatric disorders. All gave written informed consent under a process approved by the Human Research Protection Office by the Washington University School of Medicine.

### 2.2 HD-DOT Imaging System and Data Acquisition

We used a high-density, relatively large field-of-view, continuous wave, diffuse optical tomography system for data acquisition. The instrument consists of a flexible imaging cap that holds 96 source and 92 detector locations. Using fiber optic bundles, source locations are illuminated by continuous-wave light-emitting diodes. Light emitted from the head and collected by optical fibers at detector locations is detected by avalanche photodiodes (Hamamatsu C5460-01). Hemoglobin spectroscopy is enabled using two wavelengths (750 and 850 nm). The detected light signal is digitized by dedicated 24-bit analog-to-digital converters (MOTU HD192),^33^ which have high dynamic range ($>10^{6}$) and low crosstalk ($<10^{-6}$). The dynamic range allows the detection of light from multiple source-detector distances (e.g., first through fourth-nearest neighbors are separated by 13, 30, 39, and 47 mm, respectively). This array provides more than 1200 usable source-detector measurements at a 10-Hz full-field frame rate. Full details on this system have been previously described.^13^

**Fig. 1:**
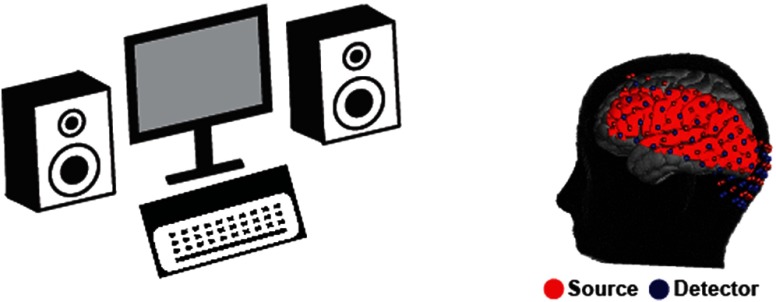
Schematic view of HD-DOT experimental setup, subject position, and imaging cap structure. Shown in red is group field-of-view overlaid on a cortical surface view of the MNI atlas.

Subjects were seated in an adjustable chair in a sound-isolated room facing a 19-in. LCD screen (located 1 m from subjects at approximately eye level) and two stereo speakers (each located at 1.5 m from subjects at approximately ear level). Subjects held a keyboard on their lap. The HD-DOT cap was put on the subject’s head covering occipital, temporal, and parts of parietal, motor, and frontal cortices (Fig. 1). Once the cap was placed comfortably with a good signal-to-noise ratio (the average signal for all the optodes across the cap was within two orders of magnitude and the temporal standard deviation of the signal was less than 7.5%), the exact placement of the cap with respect to the locations of anatomical landmarks on the head and face of the subject (e.g., the distance between optode location in one of the outer four corners of the imaging array and the nasion) was noted to locate the cap relative to the subject’s head, later used to generate a subject-specific finite element light model.^9^

### 2.3 Stimulus Protocol

We used 60 meaningful six-word sentences each with a subject-relative embedded clause, and reworded them to vary (1) their syntactic complexity (turning subject-relative into object-relative construction) and (2) the gender of the character performing the action: male (e.g., king and brother) or female (e.g., queen and sister).^34^ These manipulations resulted in 120 sentences with subject-relative center-embedded clauses and 120 sentences with object-relative center-embedded clauses, split evenly between male and female characters performing the action. The mean length of auditory stimuli was 1.76±0.05  s (range: 1.32 to 1.89 s). These stimuli are available in Ref. 35.

Sentences with object-relative clauses are more difficult to comprehend compared to those containing subject-relative clauses,^36^^,^^37^ typically resulting in longer response times,^38^ more errors, or equivalent performance but through increased neural activity.^12^ During the experiment, each of the 240 sentences was presented a single time and participants were asked to indicate the gender of the character performing the action (male or female) using a button-press response.

Additionally, we included unintelligible speech trials (noise) as a control condition. The noise stimuli consisted of one-channel noise-vocoded speech created by modulating white noise (lowpass filtered at 8 kHz) with the amplitude envelope of the sentence (lowpass filtered at 30 Hz). Noise vocoding removes much of the spectral detail from the sentence while retaining its temporal amplitude envelope.^39^ The code for noise vocoding (*jp_vocode.m*) is available in Ref. 40.

We presented stimuli using Psychophysics Toolbox 3,^41^ sending audio to the speakers via an external audio interface (M-Audio Fast Track Pro). We set the sound level at a comfortable listening level that did not change over the course of a session. Stimuli were presented in four separate runs, each of which contained 30 subject-relative (easy) sentences, 30 object-relative (complex) sentences, and 10 noise trials. These stimuli were presented in a pseudorandom order, with the order of conditions varied among runs but constant across subjects. Subjects were instructed to press a key with their left index finger if the person performing the action was female and a separate key with their right index finger if the person performing the action was male. A central fixation cross was displayed at the center of a gray screen; after each key press the cross was changed to an “x” to inform the subject that a response was received. This sign was on the screen during part of interstimulus interval (ISI). ISIs were pseudorandomly distributed between 2 and 10 s; the subject’s reaction time at each trial was considered as a part of the ISI of that trial. If the reaction time was over the predetermined ISI for a given trial, the next stimulus was presented immediately following a subject’s key press. One second prior to the stimulus trial the “x” was changed back to a cross to prepare the subject for listening to the next stimulus. Stimuli were presented in a pseudorandom order.

Prior to the experiment, subjects were given a short practice session containing 24 trials (eight trials per condition) to explain the instructions and ensure they were performing the task correctly. None of these sentences was presented in the actual experiment. Participants’ average accuracy was greater than 97% for both subject-relative and object-relative sentences and did not differ between conditions.

### 2.4 Data Preprocessing

Volumetric measurements of hemodynamic activity were obtained from standard HD-DOT preprocessing of raw detector data.^42^ HD-DOT preprocessing includes denoising by superficial signal regression and bandpass filtering (0.02 to 0.5 Hz) of ratiometric changes in light levels, followed by reconstruction of hemodynamic-related changes in the absorption coefficients within the imaging field of view (FOV) and spectroscopy to estimate changes in the concentration of regional oxyhemoglobin and deoxyhemoglobin (ΔHbO and ΔHbR, respectively). Hemodynamic signals were then downsampled to 1 Hz. For image reconstruction, we used individuals’ head models (derived from participants’ T1- and T2-weighted MRIs) to generate subject-specific forward light propagation models.^9^ For each participant, the reconstructed image was then normalized to Montreal Neurological Institute (MNI) 152 atlas space using a linear affine transformation, concurrently resampling to a voxel size of 3×3×3  mm. Due to the cap fitting on a variety of head sizes and shapes, the FOV measured within each subject varied across participants. For this study, the group FOV includes only voxels sampled with acceptable sensitivity in all subjects (Fig. 1, red area on the cortex). To find these voxels, we calculated a flat field reconstruction [reconstruction of an image where the absorbance change is assumed to be equal throughout the FOV (i.e., all the voxels)^43^] and included only voxels whose reconstructed value was within two orders of magnitude of the maximum. The group FOV contains $∼700\;{\mathrm{cm}}^{3}$ of head volume, covering occipital cortex and aspects of parietal, temporal, motor, and prefrontal cortices.

For the cortical surface representation of results, volumetric results are mapped onto the cortical gray matter midthickness surface of MNI152 atlas extracted using FreeSurfer software (version 5.1.0, Martinos Center for Biomedical Imaging, Massachusetts General Hospital).^44^

### 2.5 Timeseries Analysis

Here, we focused our analysis on oxyhemoglobin signal because (1) ΔHbO signal offers a higher contrast-to-noise ratio compared to ΔHbR and (2) we found that response maps based on ΔHbR signal are generally consistent with ΔHbO results.^12^^,^^42^ Prior to effective connectivity analysis, a standard general linear model (GLM) approach was used to localize the responses to each stimulus. Five conditions were included in the design matrix: subject-relative sentences, object-relative sentences, noise trials, left button presses, and right button presses. Auditory stimuli were modeled as events with 2 s duration and button presses as events with 0 s duration. Each event onset was convolved with a canonical HRF to produce a task-dependent reponse (TDR) model (predicted hemodynamic response). The canonical HRF was a double-gamma function with temporal properties matching the general features of the oxyhemoglobin response (averaged across all conditions and subjects) in primary auditory cortex: delay time of 2 s, time to peak of 7 s, and undershoot at 17 s. For each subject, we combined data for all four runs using a fixed effects analysis. Linear contrast maps including “sentences > baseline” and “complex sentences > easy sentences” were generated and statistical z-value maps for each contrast were calculated. We assessed group-level response maps using random effects analyses of these contrast maps. Group maps were thresholded at p<0.001 (voxelwise) and corrected for multiple comparisons using a nonstationary cluster analysis technique at p<0.05.^45^^,^^46^

### 2.6 Effective Connectivity Analysis: Generalized Psychophysiological Interaction Analysis

To obtain effective connectivity maps, we used a linear model with three types of regressors: one regressor per condition to model the response to the main effect of that condition (equivalent to the regressors used in GLM for estimating TDR maps), one regressor to model task-independent connectivity (TIC) to a selected seed region (equivalent to the resting-state connectivity), and one regressor per condition to model the task-dependent connectivity (TDC) (i.e., effective connectivity) to the seed region during that condition (the PPI term). A TDC regressor models hemodynamic activity in terms of interaction between the task and the influence of the seed location. Hence, significant effects for a TDC regressor indicate brain regions in which the influence of (connectivity to) the seed region is modulated by the task. The TDR model ($X^{\mathrm{TDR}}$) was built by convolving the time trace of each stimulus onset (noise, subject-relative, and object-relative sentences) with the canonical HRF. The hemodynamic activity (ΔHbO signal) from each seed region ($y_{s}$) was used as a regressor for TIC. We used the PPI analysis method^19^^,^^20^ to model the TDC ($X^{\mathrm{TDC}}$)

$$y_{j}(t)=X^{\mathrm{TDR}}(t)\beta_{j}^{\mathrm{TDR}}+y_{s}(t)\beta_{j}^{\mathrm{TIC}}+X^{\mathrm{TDC}}(t)\beta_{j}^{\mathrm{TDC}}+e_{j}(t),$$ (1)

$y_{j}(t)$ is the hemodynamic activity at time t and location j and $\beta_{j}^{\mathrm{TDR}}$, $\beta_{j}^{\mathrm{TIC}}$, and $\beta_{j}^{\mathrm{TDC}}$ are fitting parameters for each regressor for the location j, and $e_{j}(t)$ is the residual signal at that location. Further details about how we built $y_{s}$ and $X^{\mathrm{TDR}}$ are found in the following sections.

### 2.7 Effective Connectivity Analysis: Selecting Seeds

**Fig. 2:**
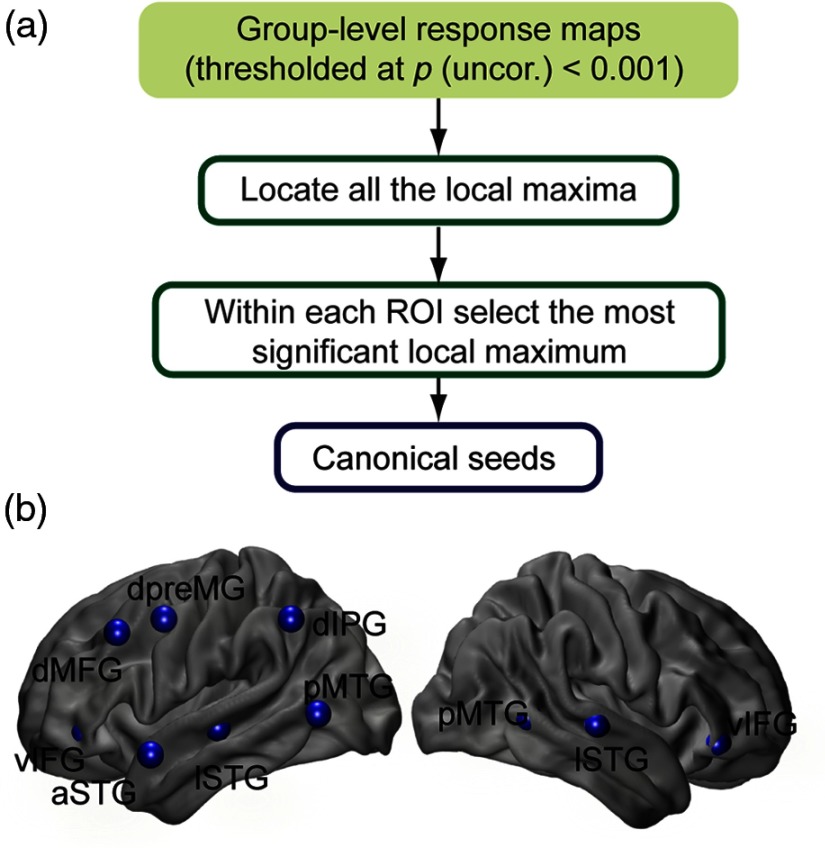
(a) Procedure of selecting canonical seeds. (b) Seeds at 10 nonoverlapping ROIs located at posterior middle temporal gyrus (pMTG), lateral superior temporal gyrus (lSTG), ventral inferior frontal gyrus (vIFG), anterior superior temporal gyrus (aSTG), dorsal middle frontal gyrus (dMFG), dorsal premotor gyrus (dpreMG), and dorsal inferior parietal gyrus (dIPG) were selected for effective connectivity analysis.

The first step in conducting a PPI analysis is defining one or more seed ROIs. We selected 10 nonoverlapping ROIs based on their known roles in speech processing including posterior and anterior parts of temporal cortex, dorsal and ventral prefrontal cortex, and premotor and inferior parietal cortex.^12^^,^^34^^,^^47^ The locations of seeds within these ROIs were specified on the basis of significantly activated (at voxelwise p<0.001 uncorrected) local maxima on group maps of auditory speech processing (sentences > noise contrast) and syntactic complexity processing (complex sentences > easy sentences contrast) (Fig. 2). Coordinates of these local maxima are given in Table 1. Seeds were defined as 6-mm-diameter spheres centered at these points. We refer to these seeds as canonical seeds because they are based on the group results rather than individual results.

**Table 1:** ROIs and coordinates of centers of canonical seeds.

Region		Center coordinates		
		x	y	z
Left				
	Dorsal premotor	−61.5	3.0	45.5
	Dorsal middle frontal	−46.5	24.0	39.0
	Dorsal inferior parietal	−55.5	−54.0	45.0
	Lateral superior temporal	−52.5	−21.0	−3.0
	Posterior middle temporal	−55.5	−66.0	3.0
	Anterior superior temporal	−67.5	9.0	−15.0
	Ventral inferior prefrontal	−40.5	39.0	−6.0
Right				
	Lateral superior temporal	67.5	−21.0	0.0
	Posterior middle temporal	64.5	−57.0	3.0
	Ventral inferior prefrontal	52.5	27.0	−9.0

Variability in response maps across individuals motivated us to investigate the use of subject-specific seeds in the effective connectivity analysis. To identify these seeds, all the local peaks in individual maps were found and among them the closest peak to each canonical seed was located. If an individual participant’s peak was within 18-mm Euclidean distance of the canonical seed, the subject-specific seed was used; otherwise, the canonical seed location was used. 18-mm threshold is selected to be a couple of voxels larger than the FWHM of estimated point spread function for our imaging array (which is ∼14  mm^48^). We chose this approach to ensure that every participant had a seed region, and that there was a reasonable amount of spatial consistency across participants.

### 2.8 Effective Connectivity Analysis: Generating Task-Dependent Connectivity Regressors

In a PPI analysis, a task-dependent connectivity (TDC) regressor is constructed by multiplying the signal at a seed region (physiological component) by a vector representing the task condition (psychological component). In this study, we took two approaches to model the TDC regressors.

**Fig. 3:**
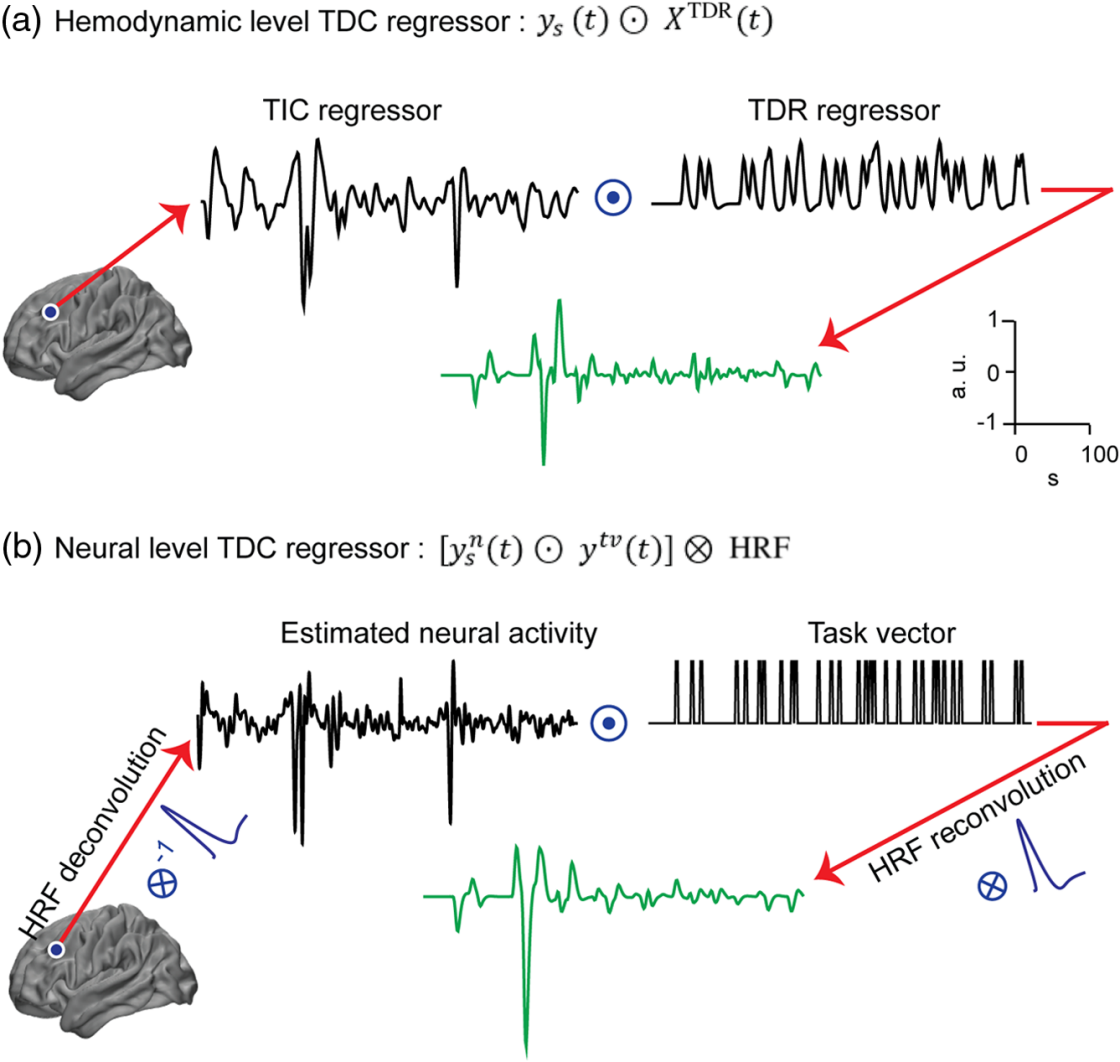
Procedure for making the TDC regressor (green curves) for PPI analysis. (a) At hemodynamic level: TDC regressor is simple componentwise multiplication (⊙) of the signal at seed location [TIC regressor, $y_{s}(t)$] and response model to task [TDR regressor, $X^{\mathrm{TDR}}(t)$]. (b) At neural level: TDC regressor is built by first deconvolving HRF from the signal at seed location [TIC regressor, $y_{s}(t)$] and multiplying the result [neural activity, $y_{s}^{n}(t)$] into task vector (vector of ones and zeros, one for when stimulus/task is on) and reconvolving the result with the same HRF. The floating scale on the right side shows the scale of signals in x and y axes.

In the first approach, we modeled interactions at the hemodynamic signal level, so the TDC regressors were built by simply multiplying the signal at the seed location to the TDR model [Fig. 3(a)]

$$X^{\mathrm{TDC}}(t)=y_{s}(t)⊙X^{\mathrm{TDR}}(t),$$ (2)

⊙ sign denotes a componentwise multiplication.

In the second approach, we modeled the interactions in a faster frequency band (closer to a neural level) with subsequent convolution by an HRF to build TDC regressors. Because HD-DOT measures of brain activity are filtered by a slow hemodynamic response, estimating interactions at the neural level can be approached by deconvolution of HRF from hemodynamic measures, noise permitting. We used a parametric empirical Bayes formulation method^21^ along with our data-driven canonical HRF (as explained earlier) to estimate faster underlying neural activity. We then constructed $X^{\mathrm{TDC}}$ by multiplying the estimated neural activity ($y_{s}^{n}$) into the task vector ($y^{tv}$), and then reconvolved the result with the same HRF used in the deconvolution step [Fig. 3(b)]. The noise introduced by the initial deconvolution step is thus smoothed by the final convolution. The task vector for each stimulus in this study was a vector of ones (epochs of 2 s for each stimulus trial) and zeros (when the stimulus is off).

$$X^{\mathrm{TDC}}(t)=[y_{s}^{n}(t)⊙y^{tv}(t)]⊗\mathrm{HRF}.$$ (3)

### 2.9 Effective Connectivity Analysis with a Permuted Signal

In order to verify the physiological relevance of our effective connectivity results, we performed a second set of PPI analyses with a signal for the physiological component that was not related to the current task-based neural activity. To hold all the other characteristics of a real signal constant—including spectral characteristics of noise (with different origins)—we used the seed signal recorded in another run (the stimuli order was pseudorandomly altered from run to run). We used a two sample chi-square test to determine whether the connectivity results based on permuted (physiologically irrelevant) signal follow the same distribution function as those based on real (physiologically relevant) signal or they are drawn from a different distribution function.

## 3 Results

**Fig. 4:**
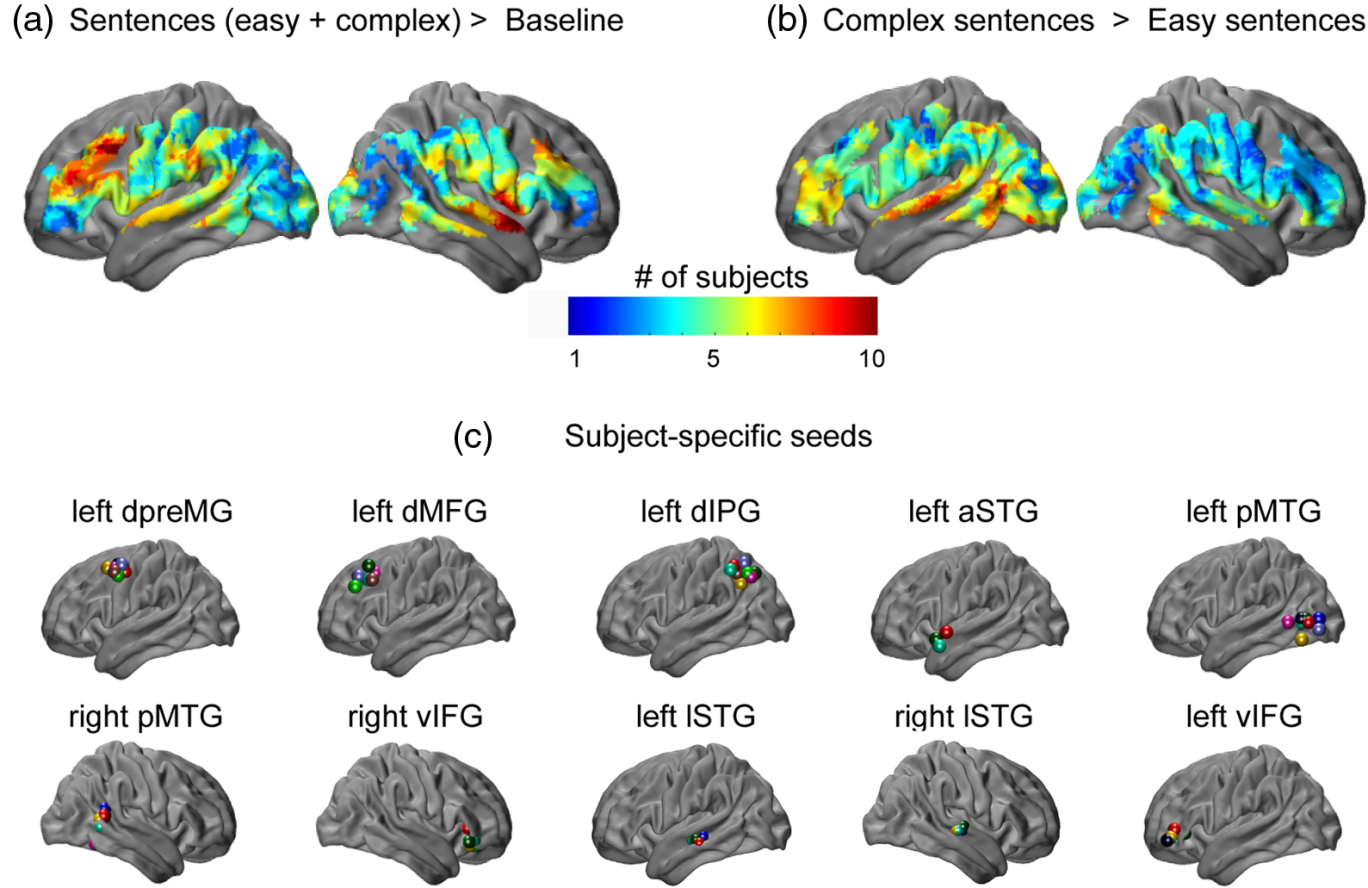
Between-subject variance in speech processing maps. Map of overlap between individuals’ significant responses (thresholded at p<0.05 uncorrected) during (a) general auditory sentence processing task (all sentences > baseline) and (b) syntactic complexity processing (complex sentences > easy sentences). The colorbar shows the number of subjects with above threshold response at a given location of cortex. (c) Individuals’ seeds at 10 selected ROIs.

To investigate response variability across subjects, we combined four runs of data collected per subject using fixed effects analyses and generated individual response maps of sentence comprehension and syntactic complexity processing. These maps were thresholded at p (uncorrected) <0.05 and converted to binary maps (1 if the voxel z-statistic was above 1.6 and 0 otherwise). As shown in Fig. 4, the overlap between individuals’ binary maps is relatively poor at some of the canonical seed coordinates: not everyone showed a significant response at those locations. When selecting subject-specific seeds, local peaks in individuals’ maps in the vicinity of canonical seeds were identified. The average distance between these peaks and the canonical seeds, as well as the number of subjects that had a local maximum within 18 mm, are given in Table 2.

**Table 2:** Subject-specific seed distribution. Average Euclidean distance between the location of subject-specific peaks and canonical seeds for each ROI and the number of subjects that had local maxima within 18 mm from each canonical seed.

Region		Euclidean distance (mm)	# subjects <18 mm
Left			
	Dorsal premotor	14.1	8
	Dorsal middle frontal	17.1	6
	Dorsal inferior parietal	12.1	9
	Lateral superior temporal	12.0	7
	Posterior middle temporal	14.7	7
	Anterior superior temporal	25.2	2
	Ventral inferior prefrontal	22.5	4
Right			
	Lateral superior temporal	15.8	5
	Posterior middle temporal	18.4	5
	Ventral inferior prefrontal	23.1	4

**Fig. 5:**
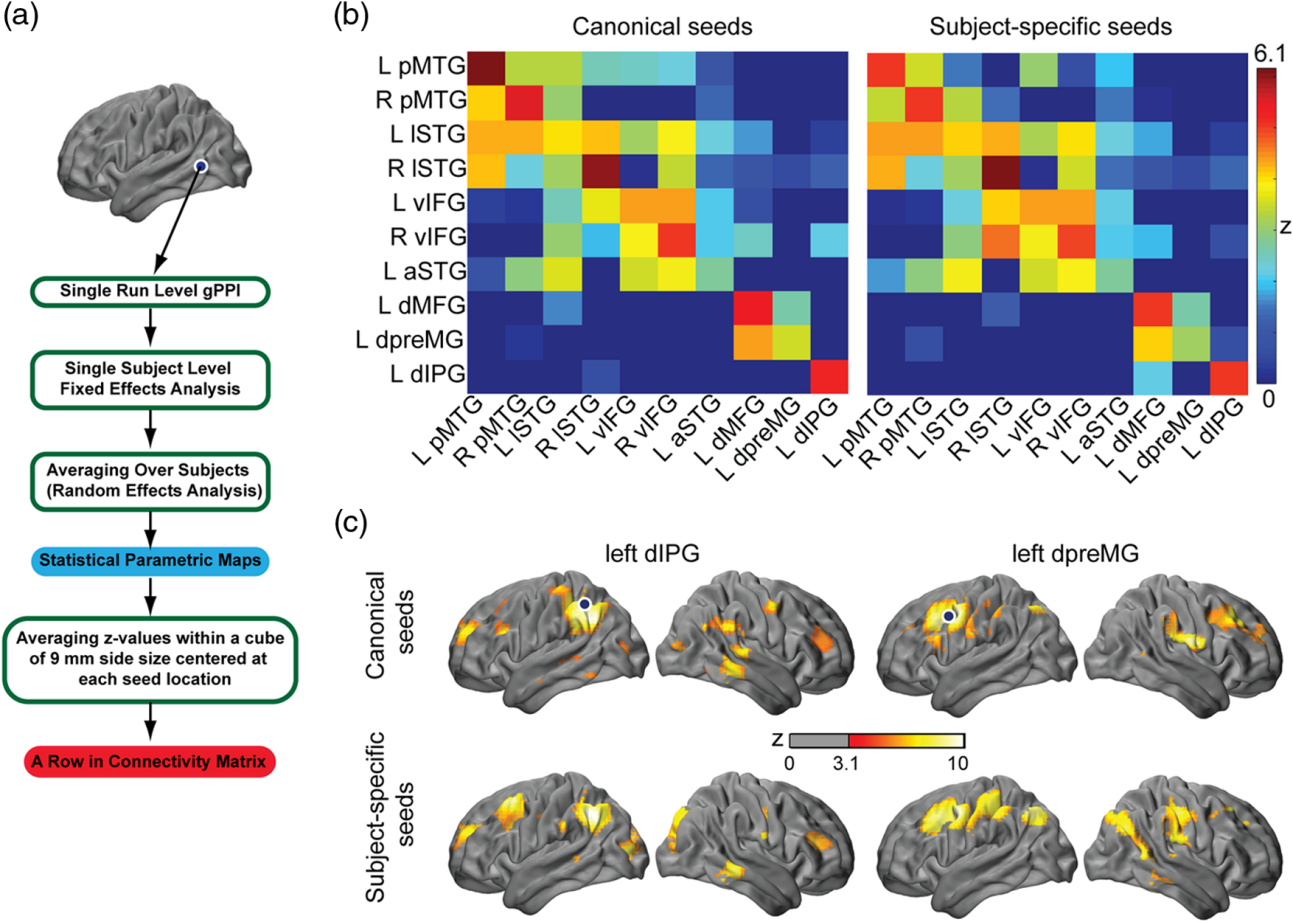
(a) Procedure for making a row in the seed-to-ROI connectivity matrix. (b) Seed-to-ROI matrices for TIC maps with canonical and subject-specific seed sets. (c) TIC maps for seeds at left dIPG and left dpreMG based on canonical and subject-specific seed sets. z-maps are thresholded at voxelwise p<0.001 (z=3.1) and (corrected) cluster significance threshold of p<0.05. These results are based on oxyhemoglobin signal changes (ΔHbO).

In order to assess the effect of using subject-specific seeds versus canonical seeds, we compared TIC maps generated with both sets of seeds. The TIC regressor ($y_{s\;}$) was built by averaging ΔHbO signal within the seed region (i.e., across voxels intersecting a 6-mm-radius sphere centered at the seed location). To summerize the resulting connectivity patterns, we calculated a seed-to-ROI connectivity matrix. For each TIC map, we averaged the z-values from thresholded group maps within cubes of 9 mm per side centered at each canonical seed coordinate; these measures made a row in the connectivity matrix [Fig. 5(a)]. Note that these connectivity matrices are not symmetrical because they are not presenting direct seed-to-seed correlation coefficients but rather results of different gPPI analyses. The seed-to-ROI connectivity matrix with both seed sets (canonical and subject-specific) looked relatively similar (correlation coefficient r=0.91) [Fig. 5(b)]. TIC maps for left dorsal inferior parietal gyrus (dIPG) and left dorsal premotor gyrus (dpreMG) seeds that had the highest number of subject-specific seeds (9 and 8, respectively) are shown in Fig. 5(c).

**Fig. 6:**
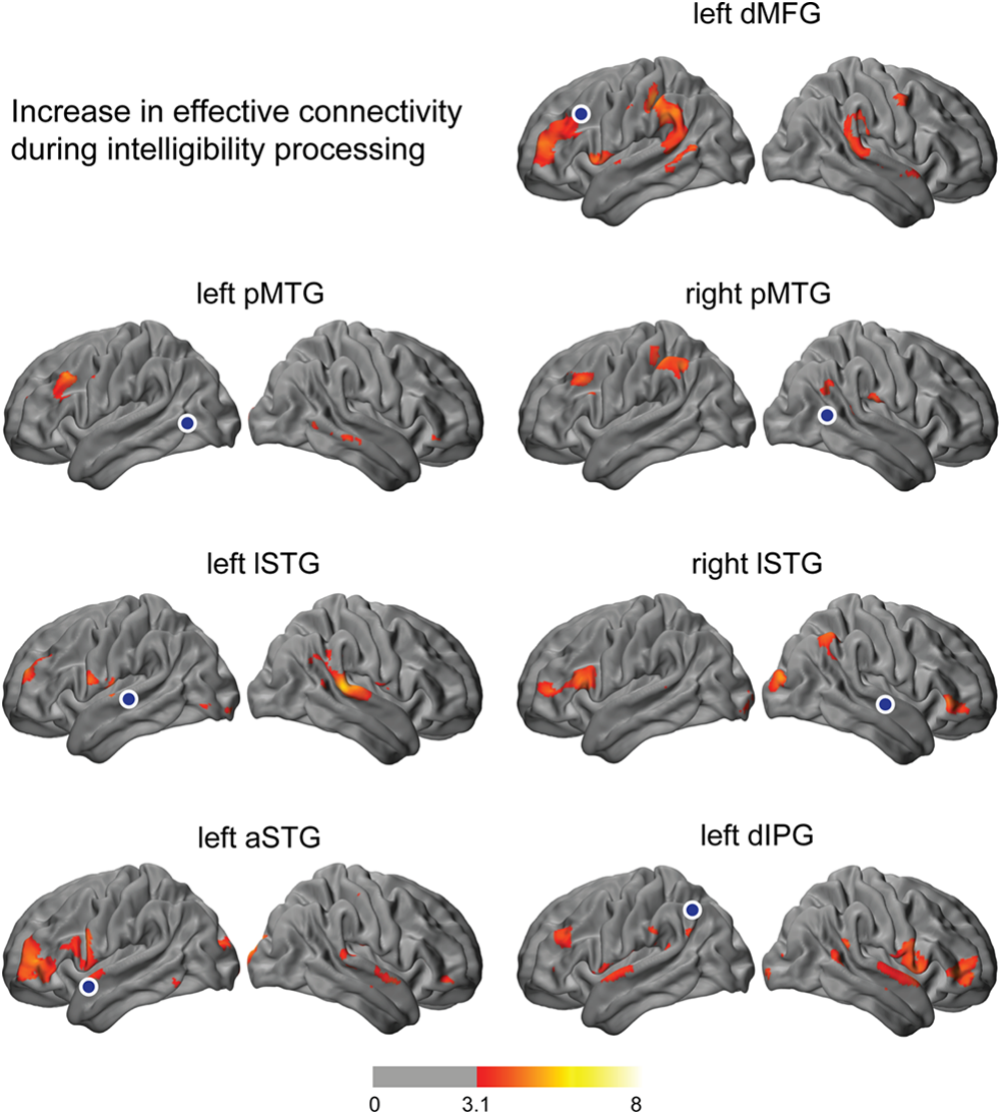
Modulatory effect of speech intelligibility on interregional connections. These maps highlight brain regions with significant increase in effective connectivity to the indicated seed locations (shown by blue circles) during sentences as compared to noise. z-maps are thresholded at voxelwise p<0.001 (z=3.1) and (corrected) cluster significance threshold of p<0.05. These results are based on oxyhemoglobin signal changes (ΔHbO).

Next, we deconvolved the canonical HRF from seed signals to incorporate the estimated neural signals in effective connectivity analysis and then we looked at how task demands influence the effective connectivity within the speech network. The effect of speech intelligibility on TDC patterns was investigated by comparing TDC maps for the sentence condition with those for the noise condition. Results showed that intelligibility significantly increased the effective connectivity between left frontal lobe and several cortical areas including both left and right posterior middle and lateral superior temporal gyri, and left anterior superior temporal and left dorsal inferior parietal gyri. In addition, intelligibility modulated the connections between left angular gyrus and left middle frontal gyrus (Fig. 6). Intelligibility did not have a significant effect on the connectivity between ventral inferior frontal gyri and the rest of the brain, nor did it affect the connectivity between left dpreMG with other regions in the brain.

**Fig. 7:**
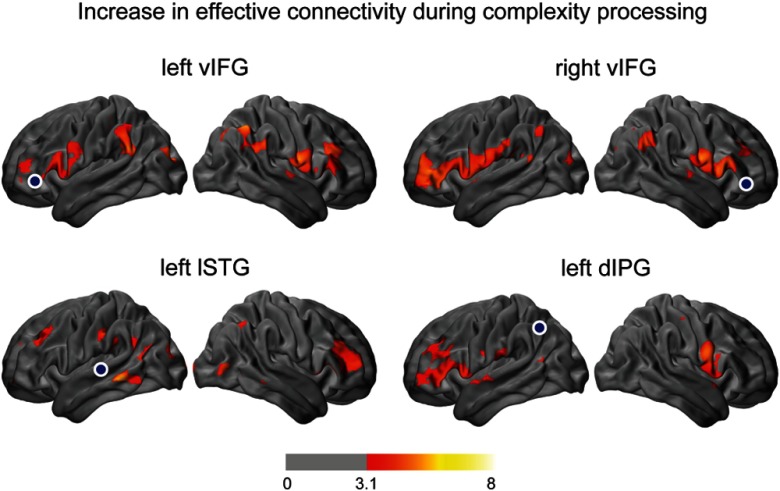
Modulatory effect of syntactic complexity on interregional connections. Statistical parametric maps showing regions for which effective connectivity to the indicated seed locations (shown by blue circles) increased when listening to complex sentences compared to easy sentences. z-maps are thresholded at voxelwise p<0.001 (z=3.1) and (corrected) cluster significance threshold of p<0.05. These results are based on oxyhemoglobin signal changes (ΔHbO).

We assessed the effect of syntactic complexity on interregional connections by statistically comparing TDC maps for the complex sentence condition with those for the easy sentence condition. These comparisons show that processing complex sentences increased bilateral connectivity between ventral parts of the prefrontal cortex and also had a strong modulatory effect on the connections between left temporal cortex and right prefrontal cortex, as well as on connections between left parietal lobe and left prefrontal cortex (Fig. 7).

**Fig. 8:**
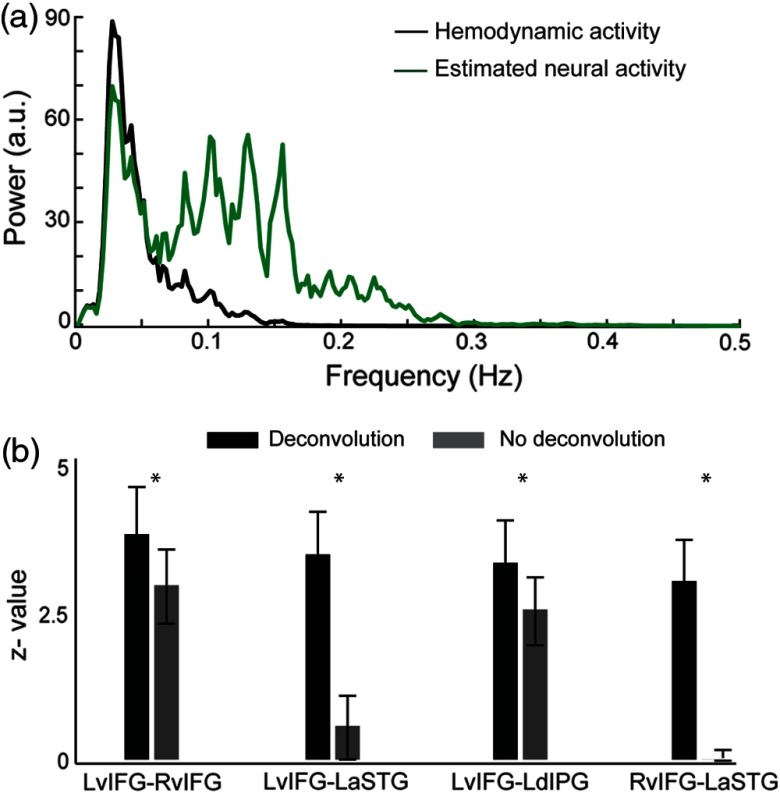
(a) Power spectra for the hemodynamic activity (black curve) and for the estimated neural activity (green curve) at a seed region. (b) Modulation of effective connectivity by task demands is significantly (p<0.05) better revealed when deconvolution is applied. These are mean (averaged across subjects) seed-to-ROI effective connectivity measures using deconvolution method (black bars) and no deconvolution method (grey bars) between left and right vIFG, left vIFG and left aSTG, left vIFG and left dIPG, and also right vIFG and left aSTG. Error bars indicate standard errors and * indicates statistical significance.

As described previously, we deconvolved the canonical HRF from seed signals to estimate the underlying neural activity. Estimated neural activity carried more high frequency components than the hemodynamic activity [Fig. 8(a)], and when incorporated in PPI models changed the effective connectivity maps. In order to statistically evaluate its effect on the maps, we looked at changes in the effective connectivity measures at the seed-to-ROI level [that summarizes maps Fig. 5(a)]. Bonferroni correction was applied to address the effect of multiple comparisons (10 comparisons per map, thus a p-value for significance at seed-to-ROI level was set to 0.005). We found significant increases in the measures of effective connectivity between several seeds and ROIs including left vIFG seed and left aSTG, left dIPG and right vIFG regions as well as those between right vIFG seed and left aSTG region [Fig. 8(b)]. For the rest of the seed-to-ROI comparisons, the effect of deconvolution of neural activity was not significant.

**Fig. 9:**
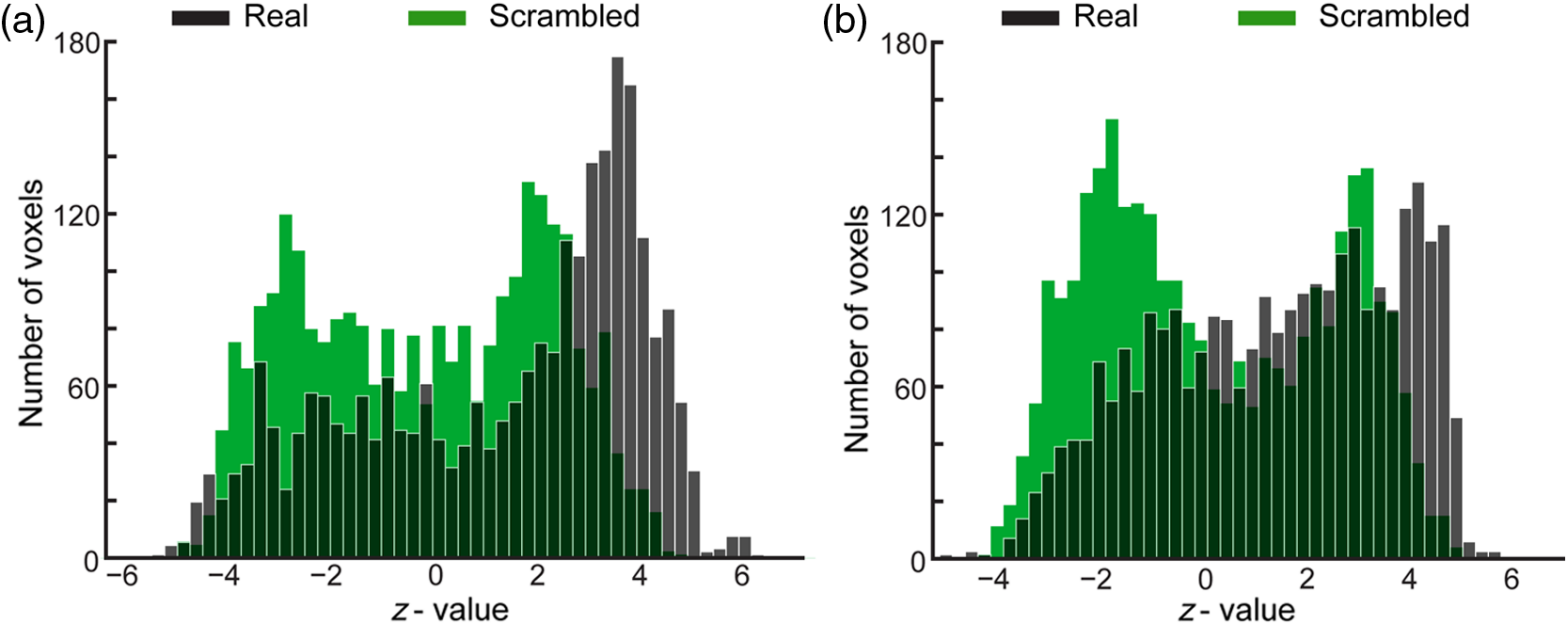
Comparing results with real and randomly assigned physiological signals. (a) Distribution of seed-to-ROI TIC measures using real (black) and scrambled signal (green), distributions were significantly different [$\chi^{2}(47)=633.6$, p<0.001]. (b) Distribution of seed-to-ROI TDC measures using real (black) and scrambled signal (green), distributions were significantly different [$\chi^{2}(43)=501.5$, p<0.001].

To examine the physiological relevance of the results, we used seed signals from different runs in the gPPI analysis. These resulted in lower connectivity values that followed a significantly different distribution as compared (using two sample chi-square test, p<0.001) to those of correct physiological signal (Fig. 9).

## 4 Discussion

In this paper, we establish the use of the gPPI analysis for HD-DOT studies of functional brain networks. We show that, as in fMRI, gPPI analyses provide useful information regarding physiologically relevant dynamic (task-dependent) connectivity among brain regions. Although several analysis approaches provided comparable results, the strongest measures of effective connectivity resulted when we included a deconvolution step in constructing the PPI model.

Our approach for estimating effective connectivity was semiexploratory in that we selected seeds based on local maxima from group maps within known speech regions. Previous neuropsychological and functional neuroimaging studies have repeatedly reported involvement of lateral portions of STG in speech perception.^49^^–^^54^ There is also strong support for the key role that pMTG plays in lexical-semantic processing^34^^,^^51^^,^^55^^,^^56^ and evidence of the role the inferior parietal cortex plays in working memory processes that are essential for speech comprehension.^57^ Additionally, multiple parts of the left frontal cortex including MFG, IFG, and premotor cortex have been repeatedly observed during speech comprehension,^47^^,^^53^^,^^58^ particularly during sentence processing.^59^^,^^60^ In addition to specifying canonical seeds in these ROI, we explored using subject-specific seeds that allow for individual variance in functional organization. Overall, a relatively small change in the TIC patterns was seen when subject-specific seeds were employed.

Effective connectivity analysis relies on modeling the interactions between tasks and brain activity. A problem arises when brain activity is measured in the form of hemodynamic response, which is delayed and smoothed by neurovascular coupling (e.g., an HRF). On one hand, we assume interactions are at the neural level, which suggests deconvolution might be helpful. On the other hand, as typically implemented, deconvolving is sensitive to noise and requires model estimations about the shape of the HRF. However, deconvolution is needed only to create the interaction term and can be followed by convolution using the same HRF. This two-step procedure minimizes the effect of model errors (same model for both deconvolution and convolution). In the data analyzed here, deconvolution of HRF seemed to enhance frequencies in the expected range for the neural activity in the speech network: as we expected to see (1) enhancement in the stimulus-locked components (i.e., between 0.1 and 0.5 Hz related to interstimulus-intervals of 2 to 10 s) and (2) slower frequencies in the higher order brain regions (related to integration of information over longer timescales in nonsensory regions^61^^–^^63^). Incorporating estimates of neural activity in the interaction model significantly strengthened the effective connectivity measures within the speech network. These included connections between left frontal lobe and several cortical areas, including both left and right posterior middle and lateral superior temporal gyri as well as left anterior superior and left dorsal inferior parietal gyri. Based on the deconvolution model, we found that speech intelligibility modulated the connections between left angular gyrus and left middle frontal gyrus. As we hypothesized, increased syntactic complexity modulated the effective connectivity among regions that we previously found to be involved in processing syntactically complex sentences.^12^ Overall, these results highlight the presence of hierarchical effective connectivity within a distributed speech-processing network.

Finally, we examined the biological relevance of the results by comparing the distributions of averaged connectivity values based on real signals with those based on scrambled signals. With the real signals, we found that distributions were leaning more toward positive values but with the scrambled signals distributions of positive and negative values were found to be relatively symmetric around 0 particularly in task-independent condition [Fig. 9(a)]. Interestingly, for the task-dependent condition [Fig. 9(b)], we found greater negative correlations with the scrambled signals. While distributions with real and scrambled signals were significantly different for both task-independent and task-dependent cases, this difference was less pronounced for the latter one in the positive values. That could be because in addition to physiological component [$y_{s}(t)$], the TDC regressor has a psychological component [$X^{\mathrm{TDR}}(t)]$ [Eq. (2)] that carries a significant amount of variance in data.

## 5 Conclusion

In this paper, we established a method for network-level study of brain function using HD-DOT. We incorporated this method in analysis of HD-DOT recordings of brain activity during different levels of speech processing and showed the modulatory effect of intelligibility and complexity on functional connectivity patterns within the cortical speech network. These advances facilitate studies of network-level brain processes using HD-DOT for a variety of tasks and populations.
